# Protection against Osteoarthritis Symptoms by Aerobic Exercise with a High-Protein Diet by Reducing Inflammation in a Testosterone-Deficient Animal Model

**DOI:** 10.3390/life12020177

**Published:** 2022-01-26

**Authors:** Sunmin Park, Suna Kang, Da Sol Kim, Ting Zhang

**Affiliations:** 1 Department of Food & Nutrition, Obesity/Diabetes Center, Hoseo University, Asan 31499, Korea; roypower003@naver.com (S.K.); tpfptm14@daum.net (D.S.K.); 2 Department of Bioconvergence, Hoseo University, Asan 31499, Korea; 20195723@vision.hoseo.edu

**Keywords:** testosterone deficiency, osteoarthritis, inflammation, oxidative stress, insulin resistance, exercise, dietary protein

## Abstract

A testosterone deficiency potentially increases osteoarthritis (OA) symptoms, and dietary protein and exercise affect them. However, their efficacy and their interactions are still unclear. We hypothesized that a high-protein diet (HPD) and regular exercise modulated OA symptoms in testosterone-deficient rats, and it was examined in bilateral orchidectomized (ORX) and monoiodoacetate (MIA)-injected rats. The ORX rats were given a 30 energy percent (En%) protein (HPD) or 17.5 En% protein (CD). Both groups had 39 En% fat in the diet. Non-ORX-CD rats (sham-operation of ORX) were given the CD and no exercise (normal control). After an eight-week intervention, all rats had an injection of MIA into the left knee, and the treatments were continued for an additional four weeks. The non-ORX-CD rats showed a significant increase in body weight compared to the ORX rats, but the ORX rats had elevated fat mass. ORX exacerbated the glucose tolerance by lowering the serum insulin concentrations and increasing insulin resistance. ORX exacerbated the OA symptoms more than the non-ORX-CD. The HPD and exercise improved bone mineral density and glucose metabolism without changing serum testosterone concentrations, while only exercise increased the lean body mass and decreased fat mass, lipid peroxide, and inflammation. Exercise, but not HPD, reduced the OA symptoms, the weight distribution in the left leg, and running velocity and provided better relief than the non-ORX-CD rats. Exercise with HPD improved the histology of the knee joint in the left leg. Exercise reduced lipid peroxide contents and TNF-α and IL-1β mRNA expression in the articular cartilage, while exercise with HPD decreased MMP-3 and MMP-13 mRNA expression as much as in the non-ORX-CD group. In conclusion, moderate aerobic exercise with HPD alleviated OA symptoms and articular cartilage degradation in a similar way in the non-ORX rats with OA by alleviating inflammation and oxidative stress.

## 1. Introduction

Osteoarthritis (OA) is a degenerative joint disease that occurs mainly in the joints of the knees, hips, and hands. OA is initiated by breaking down the joint articular cartilage and progressively changing the underlying bones [1]. OA causes pain, swelling, reduced flexibility, and stiffness, leading to joint dysfunction and disability. OA is the most prevalent joint disease worldwide. The prevalence of symptomatic OA is approximately 9.6% for men and 18% for women aged over 60 years. On the other hand, it is much higher with high variations in Asian countries—the overall prevalence in Asian countries is 20.5–68.0% [2]. The prevalence of OA has shown a rapid increase because aging is a significant risk factor, and the aged population has risen quickly in Asian countries [2].

The cause of OA is related to joint injury and overuse with increased inflammatory cytokines and matrix degradation, even though the pathophysiology remains unclear. The risk factors for OA are age, gender, obesity, and genetics. Articular cartilage mainly consists of collagen, and its injury activates collagen degradation in the articular cartilage with matrix metalloproteases (MMP) and inflammation. Primary enzymes for degenerative, progressive cartilage destruction are MMP-3 and MMP-13, contributing to OA etiology [3,4]. Middle-aged women, particularly after menopause, exhibit a higher risk for OA than men. Furthermore, low serum concentrations of androstenedione, a precursor of androgen and estrogen, are related to increasing OA in the knee and hip joints. The sex-hormone-binding globulin concentration exhibits a positive association with the OA risk, especially in the hip joint [5]. Approximately 60% of testosterone is bound to sex hormone-binding globulin (SHBG). Hence, a low SHBG indicates testosterone decline [6]. These results suggest that sex hormones and their metabolites and metabolism play a crucial role in OA development, even though their mechanism has not been investigated. On the other hand, the serum concentrations of testosterone and dihydrotestosterone are positively associated with the hip OA risk [7]. Therefore, the relationship between testosterone deficiency and OA risk remains inconsistent.

Due to chronic inflammation, a testosterone deficiency can induce cardiovascular diseases, including coronary artery disease, atherosclerosis, myocardial infarction, and stroke [8,9]. Testosterone treatments reduce inflammation in hypogonadal men [8]. Furthermore, a testosterone deficiency is positively associated with abdominal obesity, and insulin resistance and testosterone therapy suppress fat deposition [6]. A testosterone deficiency elevates their susceptibility to type 2 diabetes, metabolic syndrome, and non-alcohol hepatic steatosis. Testosterone is involved in suppressing the endoplasmic reticulum (ER) stress mechanism involved in inflammation and cell apoptosis and very-low-density lipoprotein formation and lipid export from the liver [10]. These results suggest that a testosterone deficiency potentially triggers OA by increasing inflammation, endoplasmic reticulum stress, and insulin resistance.

Despite the consequences of low testosterone concentrations, testosterone therapy is not recommended because of the increased cardiovascular events and mortality [11]. High protein intake is used to maintain or enhance muscle hypertrophy and post-exercise recovery, but it is still controversial. Moreover, the association between a high-protein diet (HPD) and testosterone status is unclear [12]. A high-fat/high-sugar/low-protein diet exacerbates myocardial tolerance in ischemia-reperfusion, decreasing the serum testosterone concentrations in rats [12]. Appropriate physical exercise is needed for OA, with minimal adverse consequences [13]. Exercise improves the OA symptoms, including pain, and alleviates them by weight loss in a systematic review. On the other hand, the type of exercise needs to be studied. Therefore, the effects of HPD and exercise on OA need to be studied.

Testosterone deficiency has been reported to accelerate OA progression [14]. Middle-aged and elderly people have increased susceptibility to OA progression, and treatment measures by changing lifestyles are needed for testosterone-deficient men. Therefore, the present study tested the hypothesis that HPD and aerobic exercise modulate OA symptoms in testosterone-deficient rats with OA and investigated the action mechanism. The hypothesis was examined in bilateral orchidectomized (ORX) and monoiodoacetate (MIA)-injected rats fed a high-fat diet. The testosterone-deficiency effects on OA were examined only in the control diet group.

## 2. Materials and Methods

### 2.1. Bilateral Orchiectomies

Fifty male Sprague–Dawley rats, eight weeks old, weighing 241 ± 16 g from DBL (Eum Sung, Korea), were acclimated in an individual stainless-steel cage in the animal facility with a controlled environment (23 °C and a 12/12 h light/dark cycle) for one week. The animal research protocol followed the National Institutes of Health guidelines for the care and use of laboratory animals (NIH Publications No. 8023, revised 1978). The Animal Care and Use Review Committee of Hoseo University approved the protocol (Asan, Korea; HSIACUC-15-03).

Male rats show sexual maturation at 41–54 days. Hence, 40 rats aged 63 days were given bilateral orchiectomies after being anesthetized with intraperitoneal injections of ketamine and xylazine (10 and 1 mg/kg BW). The rats had a mid-ventral incision in the groin to excise the spermatic cord and remove the testes; the incision was then sutured. The ORX rats were fed their assigned diets. As the normal control, male rats were given a sham-ORX operation by rubbing the testicles but not removing them. After the eighth week from the ORX or sham surgery, MIA (4 mg/50 μL saline; Sigma Co., St. Louis, MO, USA) was injected into the intra-articular cartilage through the patellar ligament of the left knee after anesthetizing them with intramuscular injections of a ketamine and xylazine mixture (100 and 10 mg, respectively) [15,16].

### 2.2. Diets and Experimental Design

The high-protein (30 En%) and high-fat (39 En%) diets were prepared with semi-purified diets based on the AIN-93 formulation [16]. The control diets (CD) contained 43.5 En% carbohydrates, 17.5 En%, protein, and 39 En% fat. The high-protein diet (HPD) increased protein content to 30% from 17.5% in CD, and carbohydrate contents decreased. CD and HPD contained the same fat contents. The HPD contained 31% energy (En%) carbohydrate, 30 En% protein, and 39 En% fat. Starch and sugar (3:2) as carbohydrates, casein as protein, and lard and corn oil (9:1) as fat were used as the primary sources of the macronutrients (CJ Co., Seoul, Korea). Water and the respective diets were freely accessible for the entire experimental period. The rats in the exercise groups walked on an uphill treadmill at 20 m/min for 30 min five days a week during the entire experimental period.

**Figure 1:**
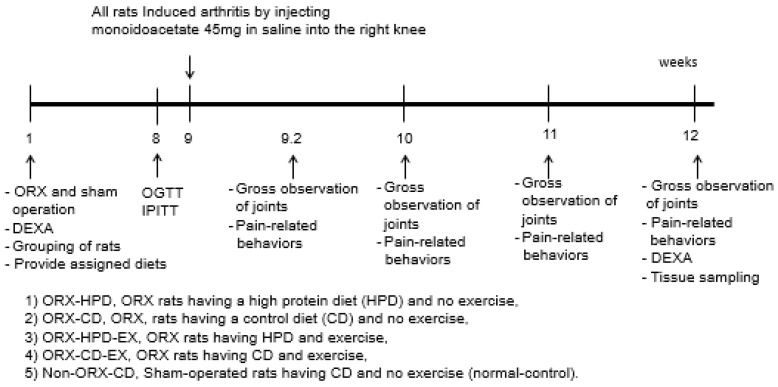
Experimental design. ORX, DEXA, dual-energy X-ray absorptiometry.

Figure 1 shows the experimental time schedules. The ORX rats were divided randomly into four groups and assigned the regime for eight weeks: (1) ORX-HPD group in ORX rats having HPD and no exercise, (2) ORX-CD group in ORX rats having CD and no exercise, (3) ORX-HPD-EX group in ORX rats having HPD and exercise, and (4) ORX-CD-EX group in ORX rats having CD and exercise. As the normal-control group, sham-operated rats were provided CD and no exercise (non-ORX-CD). Each group contained ten rats.

After eight weeks of the assigned regime, the overnight-fasted rats were given an oral glucose tolerance test (OGTT) orally administered 2 g glucose/kg body weight. The serum glucose and insulin concentrations were measured at a designated time. Two days after the OGTT, the rats with 6 h food deprivation were injected intraperitoneally with 0.75 U insulin per kg body weight, and serum glucose concentrations were measured every 15 min. The serum glucose concentrations were measured using a Glucose Analyzer II (Beckman-Coulter, Palo Alto, CA, USA). The serum insulin concentrations were analyzed using a rat ultrasensitive insulin kit (Crystal Chem, Elk Grove Village, IL, USA).

Malondialdehyde (MDA) contents in the skeletal muscles and articular cartilage were measured as an indicator of lipid peroxidation. MDA formed a colored complex in the presence of TBA, and it was detected at 532 nm using a spectrophotometer (Perkin Elmer, Waltham, MA, USA). 1,1′,3,3′-Tetraethoxypropane was used as the standard.

### 2.3. MIA-Induced OA Animal Model and OA Progression

At the ninth week from the ORX surgery, all ORX and non-ORX rats were given intra-articular injections of MIA (4 mg/50 μL saline; Sigma Co.) through the patellar ligament of the right knee, using a 26-gauge needle after anesthetizing with intramuscular injections of a ketamine and xylazine mixture (100 and 10 mg, respectively) [17].

At 2, 7, 14, and 21 days after the MIA injection into the articular cartilage of the left knee, the knee joint swelling and limping of the rats in the cages were evaluated carefully with a gross observation when they moved freely. Two trained inspectors, who were blinded to the allocations, conducted the assessments at 10 a.m. every Tuesday and classified the severity of swelling and limping as no change (0), mild (1), moderate (2), and severe (3) [14].

### 2.4. Pain-Related Behavior Assessments

The pain-related behavior of each rat was assessed using a hind paw limb weight-bearing apparatus (Linton Incapacitance Tester, Norfolk, UK) and the maximum running speed on a treadmill. Before the incapacitance test, the animals were acclimated to the apparatus for 30 min, and the weight-bearing measurements were conducted five times in each rat. Their average was used to calculate the percentage weight distribution of the right hind paw [13,14].

The maximal running speed was measured on a treadmill. The rats were acclimated to the treadmill running at 40 cm/s for 1 min, then elevated to 50 cm/s for 1 min. The treadmill velocity was then increased by 5 cm/s at 1 m intervals until the rats slid into the back of the treadmill, not to sustain the running. The maximum running speed was designated as the fastest speed that could be maintained for 20 s.

### 2.5. Body Composition

After each rat was anesthetized, the bone mineral density (BMD), lean body mass (LBM), and fat mass were assessed by dual-energy X-ray absorptiometry (DEXA; Norland pDEXA Sabre; Norland Medical Systems Inc., Fort Atkinson, WI, USA) at the beginning and end of the experiment periods. After the last DEXA, blood was collected from the vena cava after sacrifice. Epididymal fat was excised and weighed and considered the visceral fat mass. The serum testosterone and TNF-α levels were measured using ELISA kits (Enzo Life Sciences, Farmingdale, NY, USA). The lipid profiles in the blood were assessed using the calorimetry kits from Asan Pharmaceutical (Seoul, Korea).

### 2.6. mRNA Gene Expression from the Articular Cartilage by Real-Time PCR

After 21 days of the MIA injection, the rats were sacrificed after being anesthetized. The articular cartilage from five rats in each group was collected, and the rest of the knee joints were made into a paraffin block. Each cartilage sample collected was frozen in liquid nitrogen and powdered with a cold steel mortar and pestle. The total RNA of the cartilage was extracted with a monophasic solution of phenol and guanidine isothiocyanate (TRIzol reagent, Life Technologies, Rockville, MD, USA) according to the manufacturer’s instructions. The cDNA from the total RNA of each rat was synthesized using a superscript III reverse transcriptase kit (Invitrogen, Waltham, MA, USA). As previously described, each cDNA transcript and the primers of the interested genes were mixed with the SYBR Green (Bio-Rad, Richmond, CA, USA) [13,14]. The primers of the gene of interest are reported elsewhere [14]. They were cartilage degradation and inflammation-related genes, such as matrix metalloproteinase (MMP)-3, MMP-13, tumor necrosis factor (TNF)-α, and interleukin (IL)-1β genes, as described previously [14]. The gene expression levels were quantitated using the comparative cycles of the threshold method [14].

### 2.7. Histopathological Analysis of the Articular Cartilage of the Knee Joints

The histology of the knee joints was assessed for narrowing the joint region, cartilage erosion, and osteophyte formation [13,14]. The paraffin-block of the knee joints was sliced into 5 µm, and the sections were stained with hematoxylin and eosin (H-E) and Safranin O fast green. The histology was measured quantitatively using the cartilage scoring system [14]. The cartilage damage was viewed on a scale of 0–5: 0, normal; 1, only in the superficial zone (minimal); 2, invasion in the upper-middle zone (mild); 3, substantial invasion into the middle zone (moderate); 4, marked invasion into the deep zone but not to the tidemark (major); and 5, full-thickness degradation into the tidemark (severe). Tibial plateau involvement and proteoglycan loss were scored on a scale of 0–5: 0, normal; 1, minimal, 2, mild, 3, moderate, and 4, severe. The two independent trained researchers blindly scored the left joint of the rats, and the average values were used for the statistical analysis.

### 2.8. Statistical Analysis

All data were evaluated statistically using SAS software version 7 (SAS Institute, Cary, NC, USA). The results are expressed as means ± standard deviations. Two-way ANOVA was used to determine the significance of dietary protein and exercise in the metabolic effects at a single time point at the end of the experiment. The significant differences among the groups were identified using Tukey’s test at *p* < 0.05.

## 3. Results

### 3.1. Body Weight Changes and Inflammation

The body weight gain and food efficiency were much lower in the ORX groups than the non-ORX-CD group during eight weeks before the MIA injection into the left knee joint induced OA. Exercise but not dietary protein affected the body weight gain and food efficiency (Table 1). The MIA injection decreased the body weight gain in all groups, but the decrease was smaller in the non-ORX-CD rats than the ORX rats. HPD significantly inhibited weight loss (Table 1). On the other hand, the food intake was similar in the groups at the 11th week. The epididymal fat mass based on body weight was similar in the ORX and non-ORX-CD groups but lower in the exercise groups than the other groups (Table 1).

The weight changes were partly involved in the serum testosterone concentrations. The serum testosterone concentrations were lower in the ORX groups than the non-ORX-CD group, but diet and exercise did not affect the concentrations. The serum TNF-α concentration, an inflammation index, increased more in the ORX group than the non-ORX-CD group, and exercise inhibited the increase in ORX rats (Table 1).

### 3.2. Body Composition and Glycogen and Triglyceride Contents in the Skeletal Muscles

**Figure 2:**
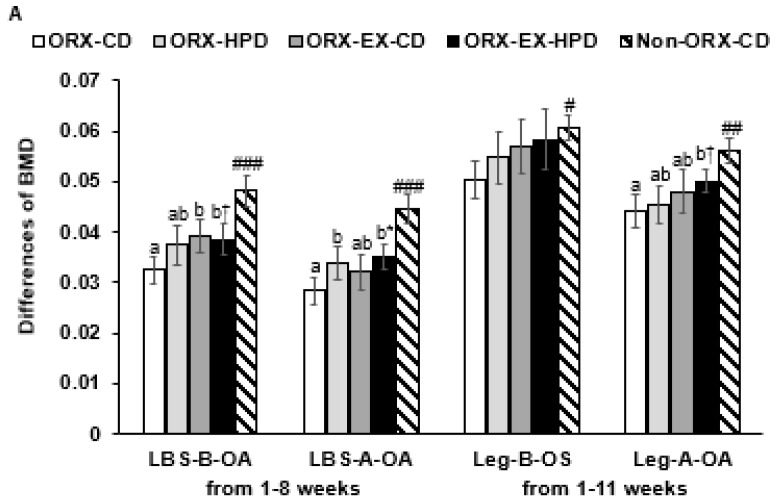

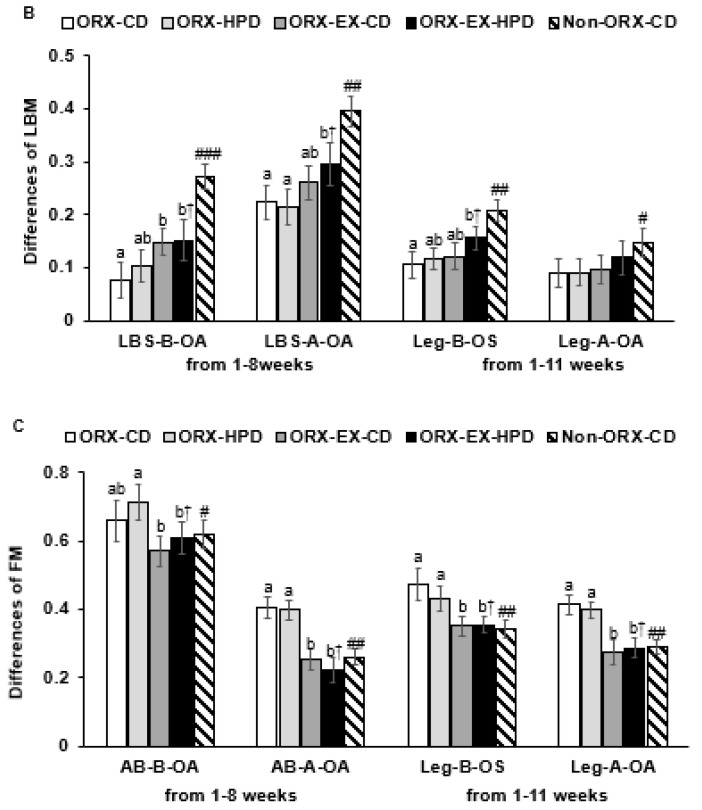
Body composition of the femur and knee at day 0 and 21 after an intra-articular injection of monoiodoacetate (MIA). (**A**) Difference of bone mineral density (BMD) in the lumbar spine, non-osteoarthritis (OA)-leg, and OA-leg; (**B**) difference of lean mass in the hip, non-OA-leg, and OA-leg; (**C**) difference of fat mass (FM) in the abdomen, non-OA-leg, and OA-leg. The orchidectomized (ORX) rats were divided randomly into four groups and assigned one of the following regimes for eight weeks: (1) ORX-HPD group in ORX rats given the high-protein diet (HPD) and no exercise, (2) ORX-CD group in ORX rats given the control (CD) and no exercise, (3) ORX-HPD-EX group in ORX rats given the HPD and exercise, and (4) ORX-CD-EX group in ORX rats given the CD and exercise. As the normal control group, sham-operated rats had CD and no exercise (non-ORX-CD). Each data point and error bar represent the mean ± standard deviations (*n* = 10). * Significantly different by diet at *p* < 0.05 by two-way ANOVA. ^†^ Significantly different by exercise at *p* < 0.05 by two-way ANOVA. ^a,b^ Different letters indicate the significant differences in the diet and exercise groups of the ORX rats at each time point, as identified by a Tukey’s test at *p* < 0.05. ^#^ Significantly different from the ORX-CD group at *p* < 0.05, ^##^ at *p* < 0.01, and ^###^ at *p* < 0.001.

The BMD was lower in the ORX rats than the non-ORX-CD rats, while the decrease in BMD was prevented with CD and exercise in the lumbar spine and left leg during 1–8 weeks and 1–11 weeks (Figure 2A). The lean body mass (LBM) decreased markedly in the hip and left leg after ORX and exercise, but HPD inhibited the decrease. After OA induction, ORX decreased the LBM, and exercise alleviated the decrease, but LBM of the left knee was not influenced by exercise and HPD (Figure 2B).

The fat mass was higher in the abdomen in the ORX group than the non-ORX-CD before OA induction, while exercise reduced the mass (Figure 2C). After OA, the fat mass was reduced in all rats, even though the changes in fat mass were similar before and after OA (Figure 2C).

Triglyceride deposition in the gastrocnemius and quadriceps muscles was higher in the ORX rats and no exercise groups, but HPD with exercise (OPX-EX-HPD) decreased the accumulation, especially in the gastrocnemius at the end of experimental periods (Table 2). In contrast to the triglyceride accumulation in the skeletal muscles, the glycogen contents were lower in the ORX rats than in the non-ORX, and HPD and exercise prevented their decrease (Table 2). Exercise and protein intake decreased MDA contents in the quadriceps, gastrocnemius, and articular cartilage (Table 2).

### 3.3. Glucose Metabolism

The serum glucose concentrations at a fasting state were higher in the ORX-CD than the other groups, while the serum insulin concentrations tended to be lower in ORX-HPD-EX than the others, but there were no significant differences (Table 1). The HOMA-IR (an insulin resistance index) was higher in the ORX group than the non-ORX-CD and HPD, and exercise lowered the HOMA-IR (Table 1).

After the oral administration of 2 g glucose/kg body weight, the serum glucose concentrations increased until 20–30 min and then decreased in all rats (Supplemental Figure S1A). The glucose tolerance of the non-ORX-CD (non-ORX) rats was similar to that of the ORX-CD-EX rats. Exercise, but not HPD, lowered the peak serum glucose concentrations in the ORX rats (Supplemental Figure S1A). The AUC of the serum glucose concentrations at the first part (0–30 min) and second part (30–120 min) during the OGTT was higher in the ORX-CON rats than the non-ORX-CON rats (Supplemental Figure S1B). Exercise lowered the AUC of the first and the second parts of the serum glucose concentrations, and HPD tended to lower the second part of the serum glucose concentrations during the OGTT. The serum insulin concentrations at 20 and 40 min were lower in the ORX-CD rats than in the non-ORX-CD rats (Supplemental Figure S1C). Exercise and HPD increased the serum insulin concentrations at 20 min in the ORX rats and decreased them at 90 min. It suggested that ORX impaired the deterioration of glucose tolerance by elevating insulin resistance and exercise protected against its deterioration.

The serum glucose concentrations at the 6 h fasting state were higher in the ORX-CD rats than the non-ORX rats, and exercise and HPD lowered the concentrations. After injecting fasted rats intraperitoneally with 0.75 IU/kg body weight insulin at 6 h, the serum glucose concentrations in the ORX-CD group were higher at all time points than those in the non-ORX-CD rats (Supplemental Figure S2A). Hence, the ORX-CD rats showed higher insulin resistance than the non-ORX-CD rats. The serum glucose concentrations decreased until 45 min and were maintained or slightly rebounded until 90 min in all rats (Supplemental Figure S2A). Exercise lowered the serum glucose concentrations markedly at 30–90 min, which was better than in the non-ORX-CD rats, and HPD decreased them slightly. The AUC of the first part was much higher in the ORX-CD rats than the ORX-HPD rats, while it was reduced with exercise. The AUC of the first part decreased in the ORX-HPD and ORX-HPD-EX rats as much as in the non-ORX-CD rats (Supplemental Figure S2B). The AUC of the 2nd part was higher in the ORX-CD rats than in the non-ORX-CD rats, and HPD and exercise lowered it. The ORX-EX-HPD and ORX-EX-CD groups reduced the AUC of the 2nd part of the IPITT more than the non-ORX-CD group.

### 3.4. Lipid Profiles

ORX increased the serum total cholesterol concentrations compared to non-ORX-CD exercise, and HPD inhibited the increase in the ORX rats (Table 2). The serum HDL concentrations were lower in the ORX rats than in the non-ORX-CD rats, while exercise and HPD and exercise suppressed the serum HDL concentrations. The serum LDL concentrations were much higher in the ORX rats than in the non-ORX-CD rats, and the increment was suppressed with HPD and exercise (Table 2). Unlike the serum cholesterol concentrations, the serum triglyceride concentrations were lower in the ORX rats than the non-ORX-CD rats, while exercise was lowered further in the ORX rats.

### 3.5. Global Observation of OA Symptoms

**Figure 3:**
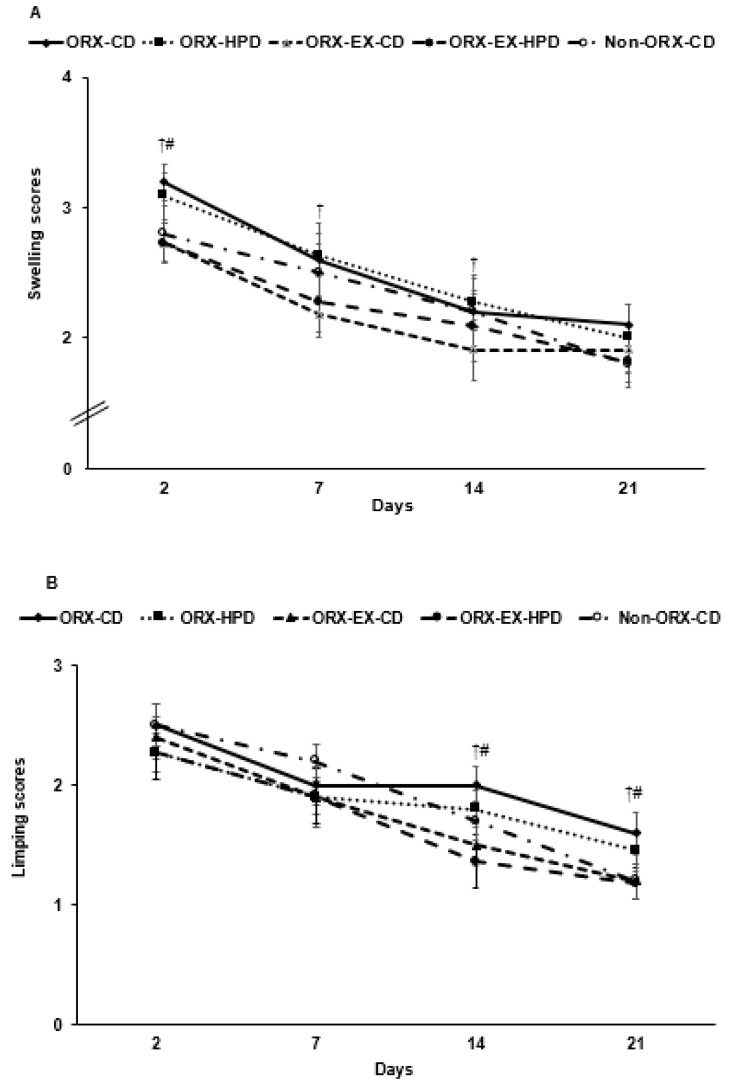

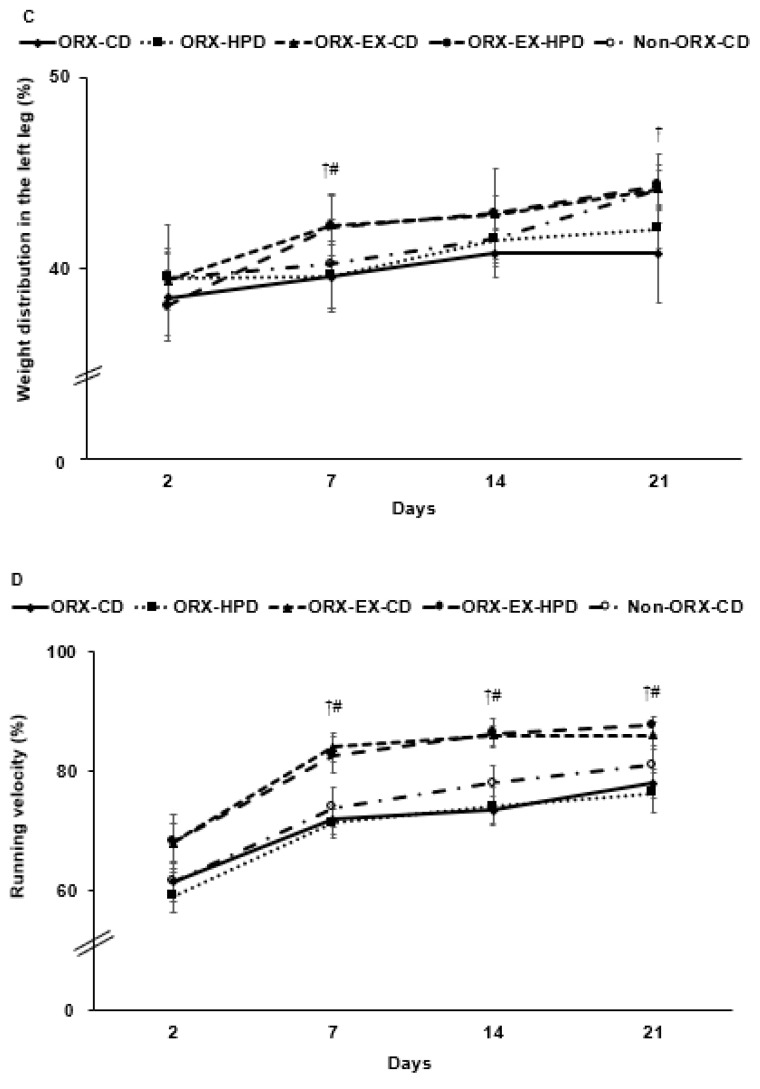
Gross observation of osteoarthritis symptoms and pain-related behaviors at 3, 7, 14, and 21 days after an intra-articular injection of monoiodoacetate (MIA). (**A**) Edema scores; (**B**) limping scores; (**C**) weight distribution between MIA- and saline-injected legs; (**D**) the maximum velocity in treadmills. The orchidectomized (ORX) rats were divided randomly into four groups and assigned one of the following regimes for eight weeks: (1) ORX-HPD group in ORX rats given the high-protein diet (HPD) and no exercise, (2) ORX-CD group in ORX rats given the control (CD) and no exercise, (3) ORX-HPD-EX group in ORX rats given the HPD and exercise, and (4) ORX-CD-EX group in ORX rats given the CD and exercise. As the normal control group, sham-operated rats had CD and no exercise (non-ORX-CD). Each data point and error bar represent the mean ± standard deviations (*n* = 10). ^†^ Significantly different by exercise at *p* < 0.05 by two-way ANOVA. ^#^ Significantly different from the ORX-CD group at *p* < 0.05.

OA symptoms, swelling and limping, were induced in all rats after the MIA injection (Figure 3A,B). The symptoms were alleviated with time in all groups. Swelling in the knee joints was alleviated seven days after the MIA injection. The ORX rats showed more swelling than the non-ORX-CD rats, and exercise improved swelling. The ORX rats limped less 14 days after the MIA injection, and exercise improved limping regardless of the dietary fat and proteins (Figure 3A,B).

Furthermore, the weight distribution in the left leg was reduced in MIA-injected (osteoarthritic) rats due to pain. The weight distribution in the left leg was higher in the ORX rats than non-ORX-CD rats while exercise regardless of dietary protein decreased it (Figure 3C). The maximum velocity on the treadmill was lower in the ORX rats than the non-ORX-CD rats and increased with exercise, but not dietary protein (Figure 3D).

### 3.6. The mRNA Expressions of Cytokines in Articular Cartilage of the Left Knee

**Figure 4:**
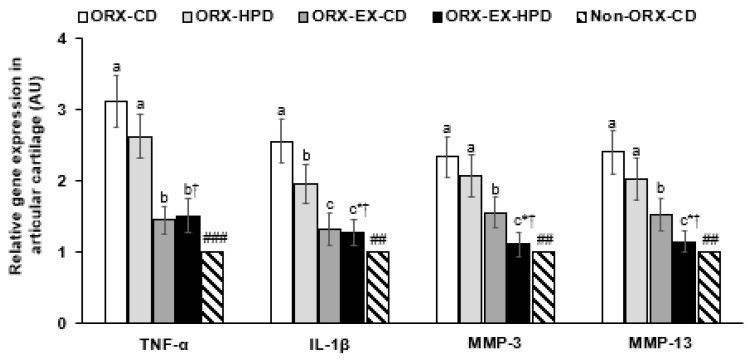
The mRNA expression of matrix metalloproteinases (MMP) and proinflammatory cytokines in the articular cartilage. The orchidectomized (ORX) rats were divided randomly into four groups and assigned one of the following regimes for eight weeks: (1) ORX-HPD group in ORX rats given the high-protein diet (HPD) and no exercise, (2) ORX-CD group in ORX rats given the control diet (CD) and no exercise, (3) ORX-HPD-EX group in ORX rats given the HPD and exercise, and (4) ORX-CD-EX group in ORX rats given the CD and exercise. As the normal control group, sham-operated rats had CD and no exercise (non-ORX-CD). Each data point and error bar represent the mean *±* standard deviation (*n* = 5). TNF-α, tumor necrosis factor-α; IL-1β, interleukin-1β. * Significant diet effect among the groups by the two-way ANOVA test at *p* < 0.05. ^†^ Significant exercise effect among the groups by the two-way ANOVA test at *p* < 0.05. ^a,b,c^ Different letters indicate the significant differences in the diet and exercise groups of the ORX rats at each time point as identified by Tukey’s test. ^##^ Significantly different from the ORX-CD group at *p* < 0.01, and ^###^ at *p* < 0.001.

TNF-α mRNA expression of the articular cartilage was higher in the ORX-CD rats than the non-ORX-CD rats, while exercise reduced it (Figure 4). IL-1β mRNA expression was higher in the ORX-CD rats than the non-ORX-CD rats, whereas exercise and HPD decreased mRNA expression. MMP-3 and MMP-13 mRNA expression levels were higher in the ORX-CD rats than the non-ORX-CD rats, and exercise inhibited the increase in the ORX rats (Figure 4).

### 3.7. Histopathological Analysis

**Figure 5:**
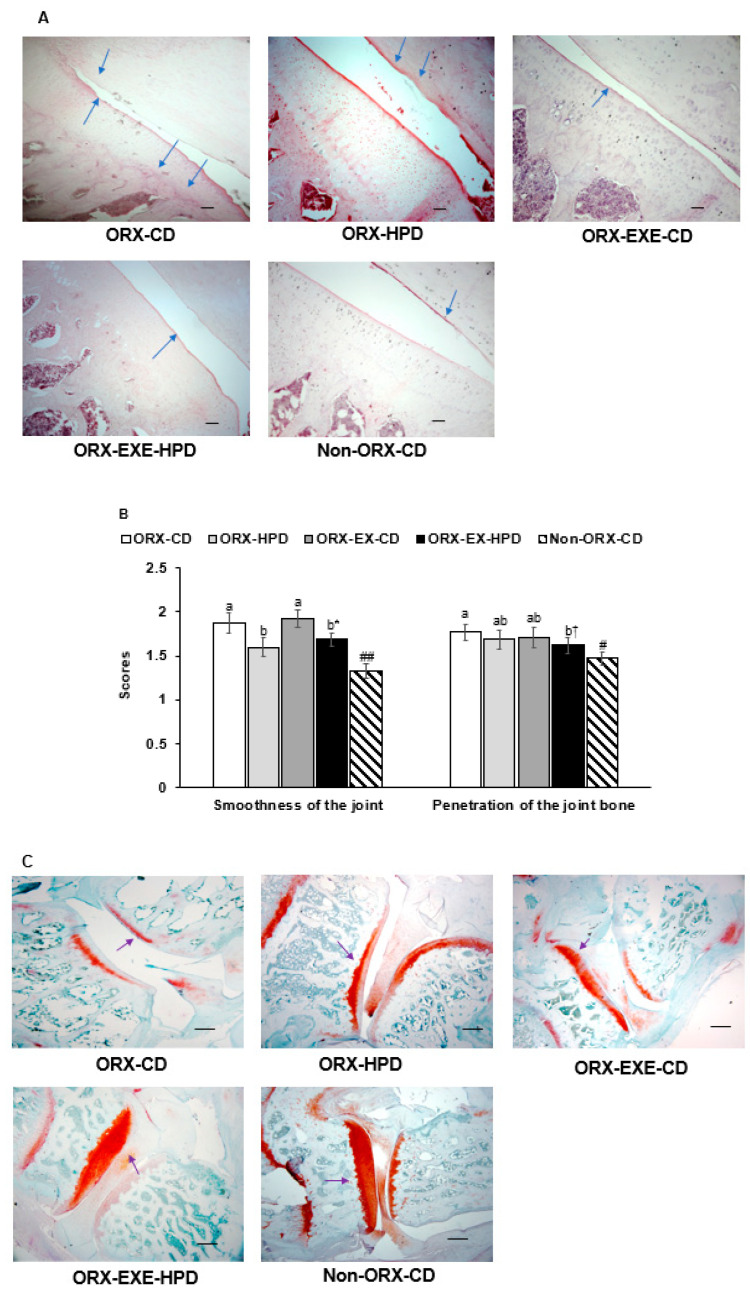

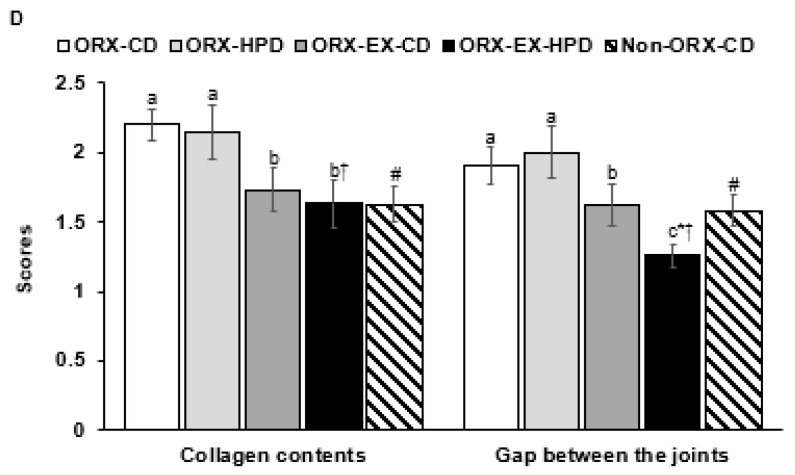
Histopathological features of the osteoarthritic knee. The articular cartilage and subchondral bone damage in hematoxylin-eosin stain (magnifying power X100 and X25). The arrows indicated the damage of the articular cartilage. (**A**) Damage of the articular cartilage and penetration of the joint bone (magnifying power X100). Scale bar represents 100 μm, and arrow indicates OA injury. (**B**) Scores of joint surface smoothness and joint bone penetration (*n* = 5). (**C**) The proteoglycan loss in the joint and gap between the joints in Safranin O fast green stain (magnifying power X25). (**D**) Scores of collagen contents and gaps between the joints (*n* = 5). Scale bar represents 500 μm, and arrows indicate collagen deposition. The orchidectomized (ORX) rats were divided randomly into four groups and assigned one of the following regimes for eight weeks: (1) ORX-HPD group in ORX rats given a high-protein diet (HPD) and no exercise, (2) ORX-CD group in ORX rats given the control diet (CD) and no exercise, (3) ORX-HPD-EX group in ORX rats given the HPD and exercise, and (4) ORX-CD-EX group in ORX rats given the CD and exercise. As the normal control group, sham-operated rats had CD and no exercise (non-ORX-CD). Each data point and error bar represent the mean *±* standard deviation (*n* = 5). * Significant diet effect among the groups by the two-way ANOVA test at *p* < 0.05. ^†^ Significant exercise effect among the groups by the two-way ANOVA test at *p* < 0.05. ^a,b,c^ Different letters indicate the significant differences in the diet and exercise groups of ORX rats at each time point as identified by Tukey’s test at *p* < 0.05. ^#^ Significantly different from the ORX-CD group at *p* < 0.05 and ^##^ at *p* < 0.01.

All rats were given an injection of MIA into the knee. Histological evaluations of H-E staining showed that the MIA injection into the left knee damaged the articular cartilage and subchondral bone in the ORX and non-ORX-CD rats. The ORX rats showed poorer histology of the knee section than the non-ORX-CD rats. (Figure 5A,B). HPD, but not exercise, alleviated the articular cartilage damage, while exercise inhibited the penetration of the joint bone in ORX rats (Figure 5A,B). In Safranin O fast green staining, collagen deposition in the knee joints was lower in the ORX rats than the non-ORX rats while exercise, but not HPD, suppressed the proteoglycan losses (Figure 5C,D). The gap between the joints was higher in the ORX rats than in the non-ORX rats, whereas exercise inhibited the joint gap (Figure 5C,D). These results suggest that exercise improved collagen deposition after the MIA injection, and HPD alleviated the joint smoothness and penetration of the joint bone.

## 4. Discussion

Although the cause of OA remains unknown, age, gender, obesity, lean body mass, and joint injury influence its incidence. Sex hormones affect the incidence of OA, with the link between estrogen deficiencies and OA risk being the central focus. On the other hand, although testosterone may increase the OA risk and its related pain, previous studies have shown that serum testosterone concentrations are inversely associated with the effusion–synovitis volume and knee pain in women [18]. Furthermore, a Mendelian randomization study showed that the serum testosterone and dihydrotestosterone concentrations are positively associated with OA and the hip replacement risk, particularly in men [5]. An inverse association between the serum SHBG concentrations and OA in men was reported [18], suggesting that a marked testosterone decline may be a risk factor for OA. Testosterone decline is a much slower process in men than estrogen deficiency in women. The present study showed that ORX deteriorated the OA symptoms, and aerobic exercise with HPD alleviated them by reducing the inflammation and insulin resistance and maintaining the LBM and BMD. Regular exercise and HPD did not significantly change the serum testosterone concentrations compared to the non-ORX group. Therefore, older men need to conduct a moderate aerobic exercise with the proper protein intake.

Aging lowers serum testosterone concentrations. The relationship between OA and serum testosterone concentrations remains controversial. Men with higher serum androstenedione concentrations are inversely associated with total knee arthroplasty caused by OA. The total testosterone concentrations are positively associated with less pain in the OA knee in men [14,19]. In a two-year follow-up study of healthy men, tibial cartilage loss is associated with the serum-free testosterone concentrations, independent of age, BMI, BMD, and tibial cartilage volume of the tibial plateau area at the baseline [20]. However, in another study, women with estrogen and testosterone deficiencies showed a positive association with low and knee effusion–synovitis and OA-related structural changes [18]. Furthermore, castrated age-matched rhesus monkeys and ORX rats develop severe joint OA [21,22]. The present study also showed that ORX rats had a lower lean body mass and BMD and a higher fat mass than non-ORX rats. Therefore, testosterone decline changes the body composition to modulate OA progression, but the mechanism is unclear.

The risk factors of OA are older age, female, obesity, insulin resistance, hyperglycemia, altered joint alignment, and articular biomechanics [1,23,24]. Furthermore, increased oxidative stress and inflammation are closely related to the development and progression of OA [25]. The fat mass and BMD are primarily consistent with the decrease in testosterone related to OA [26,27]. Serum testosterone concentrations were positively associated with lean body mass and bone mineral density, whereas they were negatively associated with fat mass and insulin resistance of men [28]. Furthermore, the relationship between OA and body composition is controversial: men with OA have a lower lean body mass and smaller bone size than those without OA [29], while men with OA have a higher BMD, BMI, and fat mass but lower lean body mass [30]. In the present study, the body weight was lower in the ORX-CD rats than the non-ORX-CD rats, but the fat mass increased, and the lean body mass and BMD decreased. ORX-CD increased insulin resistance. Therefore, obesity and insulin resistance may be crucial factors for OA, and it is involved in increasing oxidative stress and inflammation to exacerbate the OA symptoms.

Dietary modulation and exercise may improve the OA symptoms in testosterone-deficient people. A high-fat diet can promote OA development, which may be associated indirectly with increased fat mass (obesity), insulin resistance, and inflammation by high-fat diet. Calorie restriction with 12 and 30 En% fat diets results in lower body weight, but only a low-fat diet reduces the gonad fat and lessens OA symptoms in the knee joint of Hartley guinea pigs [31]. A high-fat diet with calorie restriction increases inflammation and visceral fat as much as in obese animals [31]. Therefore, body weight reduction with calorie restriction does not alleviate OA symptoms. On the other hand, the dietary composition is a crucial factor, as shown in the present study. The present study explored the effects of HPD and aerobic exercise on OA in ORX rats. The ORX-CD rats showed decreased body weight but not lower visceral fat and inflammation. Moreover, the OA symptoms were exacerbated compared to the non-ORX-CD rats. ORX-HPD rats had improved insulin resistance but did not modulate body weight, lean body mass, and fat mass compared to the ORX-CD group composed of high carbohydrates with the same fat contents. On the other hand, sufficient previous studies have shown the beneficial HPD effect on physical function and OA, especially in the elderly [32]. Intermittent fasting with HPD has been shown to alleviate OA by protecting against the lean body mass and lowering the proinflammatory cytokines, compared to the HFD in an ovariectomized animal model [16]. Therefore, the optimal dietary composition of carbohydrates, protein, and fat helps alleviate OA regardless of the modulation of the body composition.

The effects of exercise on testosterone concentrations and OA remain unclear. In a systemic review, long-term soccer training is positively associated with changes in serum-free testosterone concentrations and negatively related to the serum cortisol concentration in professional soccer players [33]. In men, the serum testosterone concentrations are positively associated with physical activity and negatively related to inflammation [34]. On the other hand, the present study did not show a positive association of serum testosterone concentrations with exercise in ORX rats, even though exercise lowered inflammation. Therefore, exercise helps reduce the increase in visceral fat, glucose intolerance, and increased inflammation in testosterone-deficient rats without changing the serum testosterone concentrations, as shown in the present study.

People with arthritis are less active than those without arthritis, even though physical activity reduces pain and improves physical function in adults with arthritis because physical activity is believed to exacerbate the clinical symptoms and pain. The most physical activity engaged in those with arthritis is walking [35]. Exercise protects against aging-associated impairment in the skeletal muscles, chondrocytes, osteoblast, and osteoclasts, contributing to the development and progression of OA [36]. Even young women with knee pain have a higher BMI and fat mass but lower thigh muscle strength and lean mass [37]. In older women in the early stages of knee OA, isometric exercise combined with electromyostimulation alleviates the pain and OA symptoms and improves knee functions and muscle strength by reducing the proinflammatory cytokines and knee function. The present study also showed that walking exercises improved pain-related behaviors, OA symptoms, inflammation status, and weight loss to decrease body fat more and muscle mass less. On the other hand, a systematic review showed that resistance training enhances the gait velocity in patients with knee OA but not knee adduction moment [38]. Furthermore, the present study showed that walking exercise reduced proinflammatory cytokine expression in the articular cartilage and the morphology of the knee joint in MIA-induced knee OA. Thus, walking exercise can help lower the fat mass and increase the lower thigh muscle strength and muscle mass in ORX rats.

In conclusion, ORX elevated the fat mass, insulin resistance, and inflammation, and moderate aerobic exercise with HPD protected against this elevation without modulating the serum testosterone concentrations. After MIA injection into the knee, the OA symptoms and pain-related behaviors were exacerbated. HPD improved the ORX-related dysfunctions and OA symptoms with moderate aerobic exercise, but HPD did not enhance them without exercise. The improvement of the OA symptoms was associated with increased collagen deposition by reducing the inflammation and degradation of the articular cartilage.

## Figures and Tables

**Table 1:** Energy and glucose metabolism.

	ORX-CD	ORX-HPD	ORX-EX-CD	ORX-EX-HPD	Non-ORX-CD
Body weight at 8th week (g)	419 ± 15.6 ^a^	422 ± 19.3 ^a^	395 ± 15.5 ^b^	404 ± 15.2 ^ab,†^	470 ± 16.4 ^###^
Food efficiency at 8th week	12.4 ± 0.75 ^a^	13.4 ± 0.96 ^a^	11.9 ± 1.06 ^b^	12.4 ± 1.11 ^ab,†^	16.0 ± 0.96 ^###^
Body weight gain at 8th week (g)	176 ± 10.9 ^a^	182 ± 16.3 ^a^	154 ± 16.9 ^b^	168 ± 16.2 ^ab,†^	229 ± 14.3 ^##^
Body weight at 11th week	392 ± 13.3 ^a^	394 ± 16.4 ^a^	372 ± 14.7 ^b^	379 ± 11.7 ^b,†^	454 ± 11.6
Body weight gain during 8–11 weeks (g)	−27.3 ± 2.1 ^a^	−28.2 ± 2.4 ^a^	−22.8 ± 1.9 ^b^	−24.9 ± 1.9 ^b,†^	−15.8 ± 1.9 ^###^
Food intake (g/day) at 11th week	14.9 ± 1.8	14.2 ± 1.7	13.8 ± 1.5	14.0 ± 1.6	14.3 ± 1.6
Epididymal fat mass (g/kg body weight)	1.69 ± 0.11 ^a^	1.62 ± 0.16 ^a^	1.51 ± 0.15 ^b^	1.54 ± 0.14 ^b,†^	1.60 ± 0.12
Serum glucose (mg/dL) at 11th week	110 ± 2.7 ^a^	102 ± 6.1 ^b^	102 ± 2.6 ^b^	103 ± 3.8 ^b,†^	101 ± 4.0 ^##^
Serum insulin (ng/mL) at 11th week	2.33 ± 0.24 ^a^	2.08 ± 0.30 ^b^	2.17 ± 0.22 ^ab^	2.07 ± 0.27 ^b,^*	2.18 ± 0.20
HOMA-IR at 11th week	4.6 ± 0.41 ^a^	3.6 ± 0.32 ^b^	3.9 ± 0.33 ^b^	3.7 ± 0.31 ^b,^*	3.9 ± 0.31 ^#^
Serum testosterone (ng/mL) at 11th week	0.83 ± 0.09	0.86 ± 0.08	0.82 ± 0.09	0.84 ± 0.08	1.98 ± 0.15 ^###^
Serum TNF-α (pg/mL)	75.7 ± 6.8 ^a^	72.7 ± 6.9 ^ab^	70.4 ± 5.9 ^b^	69.9 ± 5.7 ^b,†^	60.6 ± 6.7 ^##^

The orchidectomized (ORX) rats were randomly divided into 4 groups and assigned a regime for 8 weeks: (1) ORX-HPD group in ORX rats having a high-protein diet (HPD) and no exercise, (2) ORX-control diet (CD) group in ORX rats having control diet (CD) and no exercise, (3) ORX-HPD-EX group in ORX rats having HPD and exercise, (4) ORX-CD-EX group in ORX rats having CD and exercise. As the normal control group, sham-operated rats had CD and no exercise (non-ORX-CD). After 8 weeks of treatment, all rats had an injection of monoiodoacetate (MIA) into the left knee, and they continued the same diet for the additional 3 weeks. At the end of the experimental period, visceral fat (peri-uterine and retroperitoneum fat) mass and serum concentrations of testosterone and TNF-α, an inflammatory indicator, were measured. Values represent mean ± SD (*n* = 10). * Significantly different by HPD at *p* < 0.05 by two-way ANOVA. ^†^ Significantly different by exercise at *p* < 0.05 by two-way ANOVA. ^#^ Significantly different between ORX-CD and normal-control (non-ORX-CD) at *p* < 0.05, ^##^ at *p* < 0.01, and ^###^ at *p* < 0.001. ^a,b^ Means on the same row with different superscripts were significantly different at *p* < 0.05.

**Table 2:** Lipid profiles and glycogen and triglyceride (TG) deposition in the skeletal muscles.

	ORX-CD	ORX-HPD	ORX-EX-CD	ORX-EX-HPD	Non-ORX-CD
Serum total cholesterol	126 ± 9.3 ^a^	110 ± 8.1 ^b^	94.6 ± 7.4 ^c^	93.4 ± 7.0 ^c,^*^,††^	83.9 ± 7.5 ^###^
Serum HDL	34.1 ± 1.25 ^a^	39.1 ± 2.19 ^c^	37.8 ± 2.44 ^b^	40.6 ± 2.42 ^c,^**^,†^	42.1 ± 2.56 ^###^
Serum LDL	79.0 ± 5.64 ^a^	56.9 ± 4.12 ^b^	48.0 ± 3.34 ^c^	44.0 ± 3.21 ^d,^**^,†††^	25.8 ± 1.93 ^###^
Serum TG	64.7 ± 4.2 ^a^	70.9 ± 6.1 ^b^	49.0 ± 3.6 ^c^	48.9 ± 6.0 ^c,^*^,†††^	85.2 ± 10.4 ^##^
TG in the quadriceps (mg/g tissue)	43.8 ± 1.71 ^a^	45.8 ± 1.62 ^a^	41.2 ± 1.65 ^b^	40.8 ± 1.63 ^b,^^‡^	41.4 ± 1.64 ^#^
Glycogen in the quadriceps (mg/g tissue)	89.6 ± 2.33 ^a^	96.2 ± 2.25 ^c^	92.7 ± 1.28 ^b^	96.5 ± 1.64 ^c,^*^,†^	92.5 ± 2.35 ^#^
TG in the gastrocnemius (mg/g tissue)	37.4 ± 1.02 ^a^	36.4 ± 1.08 ^a^	30.4 ± 1.05 ^b^	28.4 ± 0.98 ^c,†^	30.9 ± 0.97 ^##^
Glycogen in the gastrocnemius (mg/g tissue)	85.0 ± 5.83 ^a^	91.0 ± 5.25 ^b^	90.0 ± 4.26 ^b^	92.3 ± 4.12 ^b,^*^,†^	90.3 ± 5.83 ^#^
Lipid peroxide in the quadriceps (umol/mg protein)	0.58 ± 0.06 ^a^	0.54 ± 0.06 ^a^	0.45 ± 0.05 ^b^	0.39 ± 0.05 ^c,^*^,†^	0.37 ± 0.05 ^##^
Lipid peroxide in the gastrocnemius (umol/mg protein)	0.55 ± 0.05 ^a^	0.52 ± 0.05 ^a^	0.46 ± 0.04 ^b^	0.40 ± 0.05 ^c,^*^,†^	0.36 ± 0.05 ^##^
Lipid peroxide in articular cartilage (umol/mg protein)	0.67 ± 0.07 ^a^	0.59 ± 0.05 ^b^	0.55 ± 0.06 ^b^	0.47 ± 0.05 ^c,^*^,†^	0.35 ± 0.05 ^###^

The orchidectomized (ORX) rats were randomly divided into 4 groups and assigned a regime for 8 weeks: (1) ORX-HPD group in ORX rats having a high-protein diet (HPD) and no exercise, (2) ORX-CD group in ORX rats having control diet (CD) and no exercise, (3) ORX-HPD-EX group in ORX rats having HPD and exercise, and (4) ORX-CD-EX group in ORX rats having CD and exercise. As the normal control group, sham-operated rats had CD and no exercise (non-ORX-CD). After 8 weeks of treatment, all rats had an injection of monoiodoacetate (MIA) into the left knee, and they continued the same diet for the additional 3 weeks. Values represent mean ± SD (*n* = 10). * Significantly different by diet at *p* < 0.05 by two-way ANOVA, and ** at *p* < 0.01. ^†^ Significantly different by exercise at *p* < 0.05, ^††^ at < 0.01, and ^†††^ at *p* < 0.001 by two-way ANOVA. ^‡^ Significant interaction between exercise and HPD at *p* < 0.05. ^#^ Significantly different between ORX-CD and normal-control (non-ORX-CD) at *p* < 0.05, ^##^ at *p* < 0.01, and ^###^ at *p* < 0.001. ^a,b,c^ Means on the same row with different superscripts were significantly different at *p* < 0.05.

## Data Availability

The authors’ raw data involved in this study will be available to any qualified researcher.

## Supplementary Materials

The following supporting information can be downloaded at: https://www.mdpi.com/article/10.3390/life12020177/s1, Figure S1: Changes in serum glucose and insulin concentrations and areas under the curve during oral glucose tolerance test (OGTT) of 2 g glucose/kg body weight (OGTT) after overnight fasting at the fifth week. Figure S2: Changes in serum glucose and insulin concentrations and areas under the curve during intraperitoneal insulin tolerance test (IPITT).
