# Supplementary material for: Protection against Osteoarthritis Symptoms by Aerobic Exercise with a High-Protein Diet by Reducing Inflammation in a Testosterone-Deficient Animal Model
Source: Life (Basel). 2022 Jan 26;12(2):177. doi: 10.3390/life12020177 (PMC8875430; doi:10.3390/life12020177)
Supplement: Supplementary file 1 [file life-12-00177-s001.zip › life-1562393-supplementary.pdf]

Supplementary figures

Fig. S1. Changes in serum glucose and insulin concentrations and areas under the curve during oral glucose tolerance test (OGTT) of 2 g glucose/kg body weight (OGTT) after overnight fasting at the fifth week.

A. Changes in serum glucose concentration measured during OGTT

B. Area under the curve (AUC) of serum glucose concentration changes during the 1st (0-50 min) and second parts (50-120 min) during OGTT

C. Changes in serum insulin concentration measured during OGTT

The orchidectomized (ORX) rats were divided randomly into four groups and assigned one of the following regimes for eight weeks: 1) ORX-HPD group in ORX rats given the HPD and no exercise, 2) ORX-CD group in ORX rats given the CD and no exercise, 3) ORX-HPD-EX group in ORX rats given the HPD and exercise, and 4) ORX-CD-EX group in ORX rats given the CD and exercise. Sham-operated rats had CD and no exercise (Non-ORX-CD) as a normal control. Bars and error bars indicated means standard deviations (n=10).

[a,b,c] Different letters indicate the significant differences in the diet and exercise groups of the ORX rats at each time point, as identified by a Tukey's test at P<0.05.

Significantly different from ORX-CD group at P<0.05.

Fig. S1A

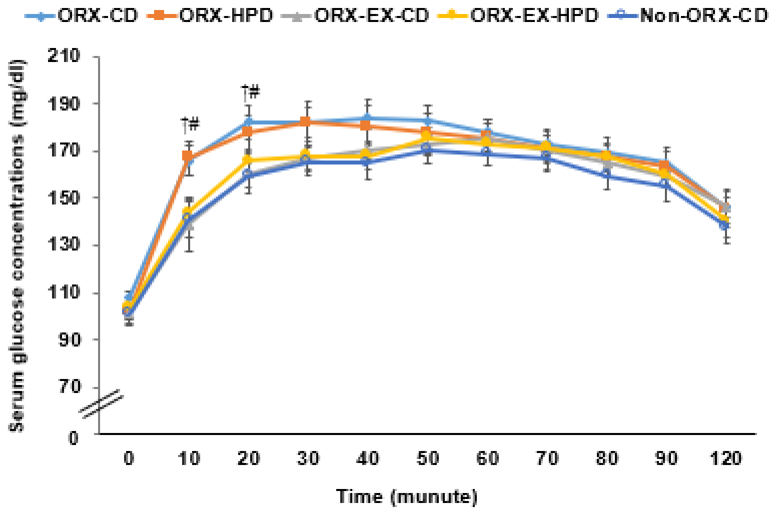

Fig. S1B

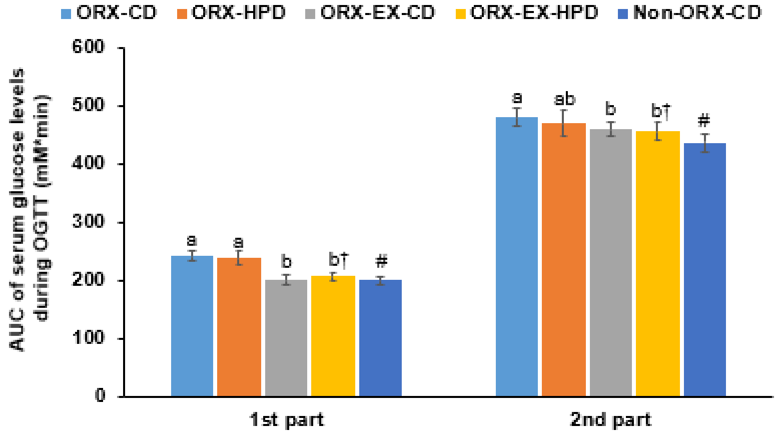

Fig. S1C

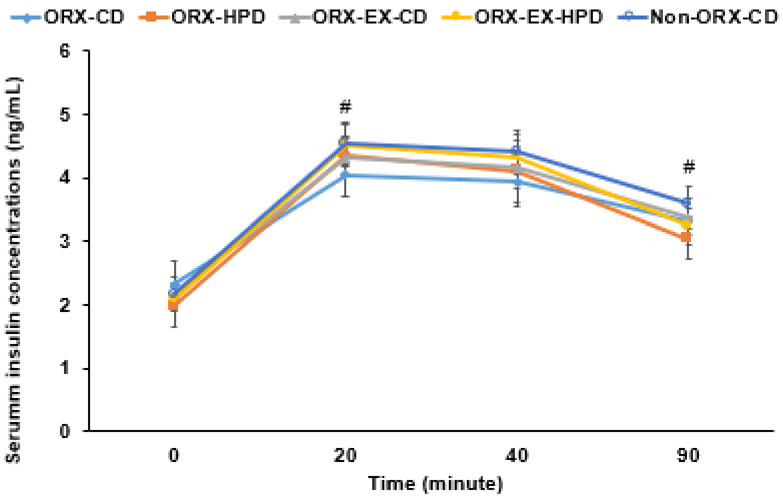

Fig. S2. Changes in serum glucose and insulin concentrations and areas under the curve during intraperitoneal insulin tolerance test (IPITT)

A. Changes in serum glucose concentration measured after intraperitoneal injection of 1 IU insulin/ kg body weight (IPITT) after 6 h food deprivation at 3 days after OGTT

B. Area under the curve of serum glucose concentration changes during the 1st (0-30 min) and second parts (30-90 min) of IPITT

Bars and error bars represent the means ± standard deviations (n = 10).

The orchidectomized (ORX) rats were divided randomly into four groups and assigned one of the following regimes for eight weeks: 1) ORX-HPD group in ORX rats given the HPD and

no exercise, 2) ORX-CD group in ORX rats given the CD and no exercise, 3) ORX-HPD-EX group in ORX rats given the HPD and exercise, and 4) ORX-CD-EX group in ORX rats given the CD and exercise. Sham-operated rats had CD and no exercise (Non-ORX-CD) as a normal control.

Each data point and error bar represent the mean standard deviations (n=10).

*Significant diet effect among the groups by the two-way ANOVA test at $P<0.05$.

† Significant exercise effect among the groups by the two-way ANOVA test at $P<0.05$.

[a,b,c] Different letters indicate the significant differences in the diet and exercise groups of the ORX rats at each time point, as identified by a Tukey's test at $P<0.05$.

Significantly different from ORX-CD group at $P<0.05$.

Fig. S2A

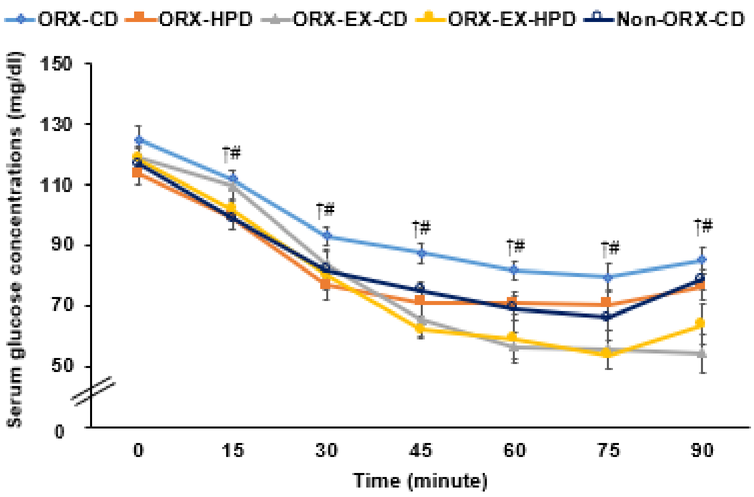

Fig. S2B

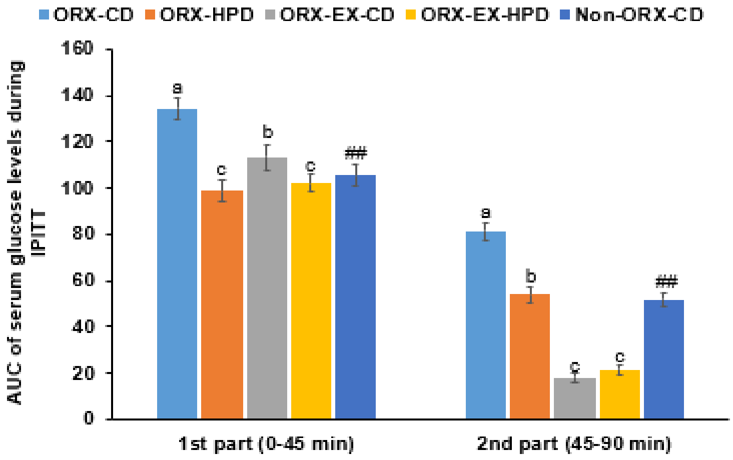

ORX-CD
ORX-HPD
ORX-EX-CD
ORX-EX-HPD
Non-ORX-CD
AUC of serum glucose levels during IPITT
160
140
120
100
80
60
40
20
0
a
c
b
c
##
a
b
c
c
##
1st part (0-45 min)
2nd part (45-90 min)
